# How do school nurses spend their time? A quantitative time study within Norwegian school health services

**DOI:** 10.1186/s12912-025-03206-6

**Published:** 2025-05-14

**Authors:** Gunhild Hustad, Marit Müller De Bortoli, Elisabeth Holm Hansen

**Affiliations:** 1 University of South-Eastern Norway, Porsgrunn, Norway; 2 Department of Child and Adolescent Health Promotion Services, Norwegian Institute of Public Health, Levanger, Norway; 3 VID Specialized University, Bergen, Norway

**Keywords:** Time study, School health services, School nurses, Public health nurses, Pupils, Children and adolescents, Guidelines

## Abstract

**Background:**

School nurses perform a unique role by providing health care to all children and adolescents. In Norway, their activities are regulated by the National Professional Guidelines for Health Promotion and Prevention Work in Health Centers, School Health Services and Youth Health Clinics. However, a large part of their workday involves independently planning and prioritizing activities. Understanding how they allocate this time is limited. Filling this knowledge gap is important, both for central authorities in their planning and staffing of the service, and for the continuous development of school health services. This study aims to describe how school nurses spend their time within the Norwegian school health services.

**Methods:**

Over a period of 10 days, 104 school nurses documented all activities spent in school health services for a cross-sectional time study. An activity list was developed that encompassed 25 activities, 10 of which were directly devoted to interactions with children, adolescents, and/or their guardians. Time was measured at 10-minute intervals and analysed via descriptive statistics.

**Results:**

Administrative work constituted the largest proportion of the registered time (22.1%), followed by individual consultations (15.9%) and meetings (10.9%). Minimal time was allocated to group activities for pupils and guardians, comprising 2.8%. The time dedicated to direct interaction with children, adolescents, and/or guardians accounted for 36% of the time, whereas the remaining 64% was spent on activities not involving direct interaction.

**Conclusion:**

This study provides insights into how school nurses spend their time within Norwegian school health services and indicates a large variation in the time spent on different tasks. Future research should investigate the reasons behind these variations and analyze the content and interventions used in various activities. Although the study is conducted in a Norwegian context, the methods for describing time usage are applicable to other health sectors and countries.

**Supplementary Information:**

The online version contains supplementary material available at 10.1186/s12912-025-03206-6.

## Background

Most countries offer some type of school health services, yet there are considerable differences in their extent, adherence to evidence-based methods, staffing levels, and implementation effectiveness [1]. School health services hold a unique and essential opportunity in promoting health and implementing primary prevention [1–4]. Given the significant portion of time children and adolescents spend in school, positive school experience is a significant determinant of young people’s quality of life [1, 5]. Additionally, the service addresses social inequality, ensuring equal healthcare access for all children and adolescents, regardless of their place of residence, gender, origin, ethnicity, or living situation [1, 4, 6–9].

School health services in Norway are mandatory and must be provided by all municipalities as governed by the Municipal Health and Care Services Act [10] and regulated by the National Professional Guidelines for Health Promotion and Prevention Work in Health Centers, School Health Services and Youth Health Clinics (hereafter Guidelines) [7]. However, municipalities maintain considerable autonomy in organizing school health services on the basis of their local conditions [6, 11]. A fundamental principle is that school health services are free of charge and easily accessible to pupils, thereby enabling all children and adolescents to independently seek health assistance when needed [7].

In Norway, school health services are mainly staffed by Public Health Nurses (PHNs), who work collaboratively with other health professionals, such as doctors and physiotherapists [7]. To qualify as a PHN in Norway, one must complete 90 study credits post baccalaureate, with the option to extending of 120 study credits for a master’s degree [12]. The Norwegian term for this advanced education in public health is “health nurses”. This title underscores the role of PHNs in emphasizing health promotion and prevention rather than focusing on disease management and treatment [7]. PHNs in Norway primarily work in three settings: health centers for children aged 0–5 years and their guardians, and/or school health services that cover from elementary throughout high school,- and/or youth health clinics for adolescents up to 25 years of age [12]. This focus on children and adolescents differs from other countries where PHNs are responsible for the entire population. Internationally, the term “school nurses” is used to describe nurses working within school health services. This term will be further used, except where the title PHN is used to describe the educational level or as a collective term that includes school nurses.

Research on Norwegian school health services suggests that staffing levels are insufficient [13], and that availability and capacity remain challenging [4, 14–16]. This situation often prohibits school nurses from having enough time to fulfill activities described in the Guidelines [17]. Consequently, school nurses report a high workload relative to the resources available to them [14, 15, 18]. As a result, important areas of school nurses’ work, including advocacy roles, personal skill enhancement, and professional development, are deprioritized [13–15]. School nurses have observed a shift in their roles, with less time allocated to preventive activities, and more time spent on individual treatment-focused role [15, 19, 20]. In a study from Sweden, school nurses perceive their role as more focused on extinguishing fires rather than preventing them from starting [21]. This analogy illustrates their experience of working in an environment characterized by insufficient resources [21]. Concerns arise, that health promotion and preventive measures are neither prioritized nor adequately resourced [14, 20].

Evaluating the effectiveness of school health services is a complex process [22]. The impacts of these activities do not show immediate results and become evident only over an extended period, making the evaluation process complex [22–24]. Studies indicate that investing in increasing school nurses’ resources is economically beneficial, as it is associated with reduced health costs [25, 26]. Nevertheless, establishing a statistically significant association between increasing resources in school health services and improved academic outcomes has been challenging [27–29].

The staffing of Norwegian school health services has been the subject of public reports, with discussions primarily revolving around the establishment of either a binding staffing norm or a recommended staffing level [6, 11]. A binding staffing is formally established in legislation. The argument against implementing this regulation is that municipalities in Norway hold a strong autonomy to organize their own services based on their needs [6]. Furthermore, the concept of a recommended staffing level refers to a minimum staffing standard. Quality and availability are the principles for staffing based on the municipality’s needs and the competencies required to provide adequate services [6, 11]. Currently, a recommended staffing level is in use, where a full-time school nurse is responsible for 300 children in elementary school (grades 1–7), or 550 adolescents in middle school (grades 8–10), or 800 adolescents in high school (3–4 years post 10th grade) [11].

The Norwegian Directorate of Health recently developed a staffing calculator, currently available for use at the middle school level to assist municipalities in planning their staffing. This calculator suggests a recommended staffing percentage on the basis of average calculations of tasks recommended for school nurses, and relative to the number of pupils [30]. However, a Norwegian report from 2020 emphasized the challenge of establishing a staffing level for a school health service that is not clearly defined. This lack of clarity makes it difficult to assess how well a recommended staffing norm aligns with actual needs [14].

Studies investigating the activities of school nurses in school health services are limited. A study titled “How do nurses spend their time in school?”, conducted in New York in 1957, presents a study similar to this one [31]. The findings from this study indicate that when school nurses are provided with assistance, they can dedicate more of their time to nursing activities. Another study that conducted a time analysis of school health services was a Swedish study from 1996. This involved 26 school nurses who registered their activities over 10 days. Nearly half of the school nurses’ time was dedicated to individual health follow-ups and preventive health measures, whereas administrative tasks consumed 24% of their time [32]. No studies have identified where school nurses register their time usage within Norwegian school health services. However, in a Norwegian report from 2023, school nurses were asked about their time allocation. According to their self-reported estimates, the school nurses indicated that they spent approximately 68% of their time on individual-focused work and 13% on other tasks, such as courses and administrative duties [16].

Studies by Glavin et al. and Schaffer et al. [33, 34] highlight the necessity of enhancing the visibility and recognition of PHNs’ contributions to public health in their respective municipalities. School nurses in school health services need to advocate for their work and the societal mandate they hold in preventive and health-promoting efforts [35, 36]. As Morse et al. [37] point out, there is a crucial need for school nurses to clarify their roles, articulate their needs, and define their future paths.

The aim of this study is to describe how school nurses spend their time within Norwegian school health services; how this aligns with the Guidelines; and to quantify the time spent in direct interactions with children, adolescents, and/or their guardians, as well as the time spent on activities not involving direct interactions.

## Methods

### Design and setting

A cross-sectional time study was conducted to assess school nurses’ activities within school health services. The study was situated in the context of Norwegian school health services, encompassing primary and secondary schools. This included elementary schools, middle schools and high schools.

### Recruitment and participants

Three counties in southeastern and central Norway, encompassing 120 municipalities, were invited to participate in the study (Fig. 1). Participation required being in a school nurse position, working either full- or part-time in school health services.

To recruit participants, invitations were distributed via the leaders of the school health services in each municipality. To ensure that all leaders in these municipalities received an invitation, contact information was verified via telephone with employees in each municipality. In May 2023, an email containing an invitation and details of the study was sent to all leaders, who then forwarded the information to their school nurses in the school health services. In addition, all municipalities received a written invitation via a post, followed by two reminders via email. Registrations were processed through a digital registration form titled; Nettskjema [38]. Within this platform, multiple background variables were digitally mapped and linked with each participant via an individual reference number.

In the email, leaders were asked to provide feedback regarding the number of school nurses in the school health services who received the invitation. The aim was to keep track of the total number of individuals invited to participate in the study. However, owing to a lack of feedback, it was not possible to determine the exact number of school nurses who received the invitation. As a result, information on the total potential participants on the basis of the number who received an invitation is missing. To ensure a sufficient response, an email was sent to all registered participants, offering them the opportunity to ask questions about the study. They were also invited to a digital information meeting to gain further details. Each participant received the necessary time recording materials either by mail as a letter, or as an email attachment. These materials included the activity list, an instruction sheet, a sample of a completed time log, and 10 blank log sheets. Additional files are included with the activity list (additional file 1) and a sample of a completed time log (additional file 2).

Written informed consent was obtained from those who expressed willingness to participate in the study, with the assurance that they could withdraw their participation at any time, until the data analysis stage. The study received approval from the Norwegian Agency for Shared Services in Education and Research on May 10, 2023, under reference number 263,032.

### Data collection

The initial focus was on developing the material for the time recording process, as no such material was found to be available. Four expert groups, each consisting of up to four participants (*n* = 14) from various municipalities, contributed to the development of the study. The participants in the expert groups worked within the school health services and had more than one year of experience. Twelve of the experts held a PHN qualification; the remaining two were registered nurses. Their primary responsibility was to contribute to the preparation of information sent to participants, the development of the time log and activity list, and to provide insights into the feasibility of the time registration in terms of effort and time consumption. They also offered suggestions on how the study could be integrated into the workday within the school health services. To establish a basis for a list of school nurses’ activity codes, the initial code list of 30 activity codes was developed on the basis of the Guidelines.

Each expert group underwent a similar process. Initially, they were presented with the existing material, which was read aloud prior to any discussion. The expert groups subsequently provided feedback for necessary revisions and adjustments. Following each session, modifications were made based on the feedback. As a result, the expert groups were presented with varying versions of the activity list. A reduction in the number of adjustments was observed with each review by the expert groups, signifying a continual refinement of the activity list and the time log.

Three school nurses with more than five years of experience working in school health services, pilot tested the time recording material for a day and provided feedback for its final refinement. The final version of the activity list included 17 codes for school nurses’ activities and 6 codes for additional activities. These additional activities included waiting, technical problems, travel and parking, break, sick leave, and vacation. An additional code was incorporated for work conducted outside the school health services, and a separate code was created for tasks not included in the activity list.

Each participant kept a daily logbook for ten consecutive days, registering activities at 10-minute intervals. Any activities that lasted less than 10 min were not registered. If an activity started or ended within a 10-minute interval, standard mathematical rounding practices were followed. To avoid misreporting, the data was registered in real time. The registration covered two workweeks (autumn weeks 44 and 45 in 2023), which were selected for being free of holidays. A concession was made for five participants who, owing to scheduling conflicts, chose two different weeks in November and December 2023.

To differentiate between various school levels, participants prefaced each code with a letter. In order to distinguish between elementary school, middle school, and high school. This procedure was required for each use of an activity code, considering that some participants were employed across different school levels. The logbooks were returned either digitally or via mail, each with an attached reference number. The data from these logs were manually entered into Excel and then transferred into SPSS. To ensure data input accuracy, 10% of entries were randomly inspected and cross- checked.

### Data analysis

Descriptive statistics were utilized to present the duration of activities in 10-minute units. The distribution of activities and the total time spent in direct interaction, either with or without children, adolescents, and/or guardians, was expressed as a percentage of the total time used. To measure the variation in time registered by each school nurse, percentage variables were established for each activity on the basis of the individual’s total time. All the data were analysed via SPSS Statistics Software version 29.

## Results

**Fig. 1:**
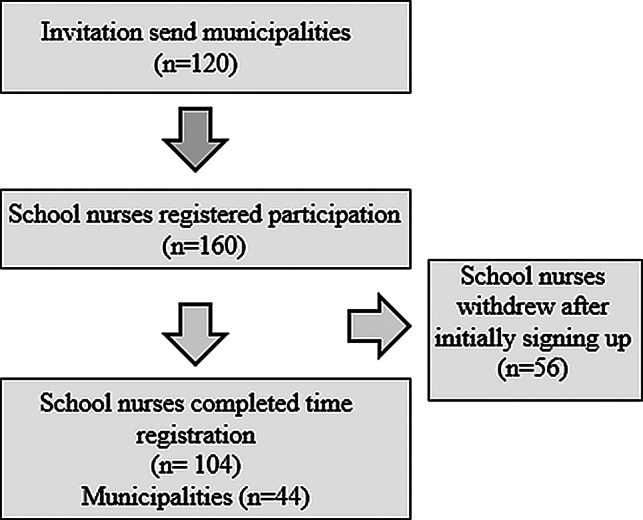
Flow diagram on recruitment and participation

### Participants

Of the 160 school nurses who registered interest in participating, 56 withdrew after initially signing up. These were given the opportunity to explain their reasons for non-participation via email: 14 withdrew due to illness, 24 cited capacity issues, and 4 withdrew due to a combination of these issues. There were 14 school nurses who did not report any reasons for their withdrawal. Consequently, the study included a total of 104 school nurses from 44 different municipalities, representing both rural and urban districts (Table 1). Furthermore, 65 participants worked solely in school health services, both full-time and part-time. The remaining 39 school nurses, in addition to working in school health services, also had responsibilities in areas such as management, or infection control, or undertook tasks involving the youngest children at health centers. The majority of the school nurses who participated worked in primary schools, with a decreasing number in higher grades. Given the standard number of employees per pupil and the number of grades, the sample is naturally skewed toward having more school nurses working in lower grades than in higher grades.

**Table 1:** Characteristics of the participants (*n* = 104)

Characteristics	*n*	%
**Age (years)**		
20–30	3	2. 9
31–40	24	23. 1
41–50	47	45. 2
51–60	25	24. 0
61+	5	4. 8
**Experience as a public health nurse (years)**		
0–2	12	11. 5
3–6	29	27. 9
7+	63	60. 6
**Experience in school health services (years)**		
0–2	16	15. 4
3–6	30	28. 8
7+	58	55. 8
**Education**		
PHN educated	96	92. 3
Working in a school nurse position educated as registered nurse	8	7. 7
**Working in education level**		
Elementary School: 1st-7th Grade	49	47. 1
Middle School: 8th-10th Grade	20	19. 2
High School: 3–4 years	10	9. 6
Working across multiple school levels	25	24. 0
**Number of schools served**		
1 school	67	64. 4
2 schools	29	27. 9
3 schools	8	7. 7
Size of municipality*		
Small	10	9. 6
Medium	28	26. 9
Large	66	63. 5

*Size of the municipality on the basis of population; data retrieved from SSB 2023: 1 = small municipality with fewer than 4900 inhabitants, 2 = medium: 5000–19,999 inhabitants, 3 = large: 20,000 or more inhabitants

### Registration and activity codes

In total, the 104 school nurses registered 999 days. However, as the study’s objective was to measure time spent on different activities within the school health services, the times registered for sick leave, absence due to vacation, and work conducted elsewhere were excluded from the results. This exclusion resulted in a variation in the registered time among the participants. A total of 41,322 ten-minute intervals were registered, equating to 861 workdays based on an eight-hour workday, considering that breaks were included in the registered data. On average, each participant recorded 397 ten-minute intervals. This translates to an average of 66 h of activity over a two-week period, or approximately 6.6 h per day.

The activity codes developed in collaboration with the expert groups aligned well with the tasks school nurses spent their time on in this study. The code assigned to represent any other tasks, intended for the school nurses to use if the existing codes were not applicable, was used minimally. Two new codes were introduced that could have been included. These were tasks related to practical activities and fire drills. However, the time allocated to these two codes was so minimal, constituting a total of five hours, that it was excluded from the results. The time spent on follow-up with PHN students did not have a specific code. However, this time was included in the activity code for courses and professional development.

Some participants used dual codes, meaning that they performed two activities simultaneously and recorded two codes. In the results, this multitasking time is divided equally, implying that one code accounts for half the time, and the other code covers the remaining half. In total, eight multitask codes were recorded, totaling 28 h of multitask-coded time. Five of these codes represented instances where breaks were taken concurrently with other tasks, whereas two of the codes indicated periods of waiting combined with other activities. The results indicate a large variation in the use of time for the different activities. Table 2 presents the total time used, measured in 10-minute blocks, distributed across all schools, both in total and as percentages, where the percentage base is the total time spent.

**Table 2:** Activities registered in 10-minute intervals and percentages

Activity Task	Elementary school		Middle school		High school		All schools**	
	10 min.	%*	10 min.	%*	10 min.	%*	10 min.	%*
Administrative tasks	5063	22. 4	2940	22. 4	1116	20. 0	9119	22. 1
Consultation	2824	12. 5	2125	16. 2	1634	29. 3	6583	15. 9
Meetings	2251	9. 9	1636	12. 5	601	10. 8	4488	10. 9
Courses and studies	1565	6. 9	1072	8. 2	351	6. 3	2988	7. 2
Collaboration guardians	1630	7. 2	495	3. 8	115	2. 1	2240	5. 4
Break	1210	5. 4	668	5. 1	283	5. 1	2161	5. 2
Collaboration school	1226	5. 4	588	4. 5	260	4. 7	2074	5. 0
Collaboration health	1176	5. 2	482	3. 7	242	4. 3	1900	4. 6
Preparation/planning	1202	5. 3	510	3. 9	185	3. 3	1897	4. 6
Vaccination	834	3. 7	705	5. 4	279	5. 0	1818	4. 4
1st grade consultation	1345	5. 9	0	0	0	0	1345	3. 3
Travel and parking	697	3. 1	332	2. 5	95	1. 7	1124	2. 7
School environment	501	2. 2	207	1. 6	60	1. 1	768	1. 9
8th grade consultation	0	0	709	5. 4	0	0	709	1. 7
Universal teaching	284	1. 3	201	1. 5	168	3. 0	653	1. 6
Individual plan	177	0. 8	116	0. 9	75	1,3	368	0. 9
Group with guardians	114	0. 5	180	1. 4	0	0	294	0. 7
Waiting and delays	122	0. 5	94	0. 7	68	1. 2	284	0. 7
Group with pupils	185	0. 8	17	0. 1	15	0. 3	217	0. 5
Technical problems	102	0. 5	48	0. 4	25	0. 4	175	0. 4
3rd grade consultation	117	0. 5	0	0	0	0	117	0. 3
**Total**	**22,625**	**100**	**13,125**	**100**	**5572**	**100**	**41,322**	**100**

*The percentage basis is the total time spent measured in 10– minute blocks

**Ranked in descending order on the basis of all schools

### Activities directly related to interactions with children, adolescents, and/or guardians

Activities involving direct interaction with children, adolescents, and/or their guardians were categorized under 10 distinct codes in the activity list. These activities covered a wide range of tasks. The activity that consumed most of this time was consultations. When the school nurse is present at the school, children and adolescents have the option to visit the school nurse’s office for both scheduled and unscheduled consultations (drop-ins). These consultations constituted 15.9% of the total time.

In addition to the consultations, three codes constitute the routine consultations offered to all pupils. The first is school- start consultations, where first-grade pupils, accompanied by their guardians, undergo a thorough health check by both a doctor and a school nurse. In the 3rd grade, the height and weight of all the children are measured. The final routine check-up is the 8th-grade health conversation, where the adolescents meet the school nurse for a dialogue about health and well-being. All these routine health consultations accounted for 5.3% of the total time.

There were two codes that involved interactions with guardians. The first activity, described as collaboration with guardians, involved conversations, phone calls, and meetings. This accounted for 5.4% of the total time. The second activity; guardian’s guidance group, accounted for 0.7% of the total time.

Another primary activity for school nurses in school health services is administering vaccinations following the Norwegian Child Vaccination Programme, which mandates five individual vaccine doses [39]. This task accounted for 4.4% of the total time. Although there are no vaccines in the Norwegian Child Vaccination Programme for high school students, school nurses still spend 5% of their time vaccinating this age group. This is explained by adolescents aged 16–19 years being offered a vaccine against meningitis, which is often administered through school health services [40].

In addition to vaccinations, there were two codes where pupils were gathered in groups. One code was assigned for universal teaching of entire classes or groups, while the other code was assigned for group sessions on the basis of specific indications. These accounted for a total of 2.1% of the school nurses’ time.

The last code related to time spent with pupils was a code for the school nurse being in the school environment. As per the instructions, this time included periods spent in the schoolyard, on school trips, and in the schools’ communal areas. Collectively, these accounted for 1.9% of the time.

In summary, all the tasks that the school nurses performed directly with the pupils, or their guardians consumed 36% of the total time that the school nurses had available for the school health services (Table 3).

### Activities not directly related to interaction with children, adolescents, and/or guardians

Activities that did not involve direct interaction with children, adolescents, and/or their guardians were categorized under 11 distinct codes in the activity list. Over a two-week period, the school nurses reported that a considerable portion of their time was dedicated to administrative tasks. These tasks, defined in the instructions, encompassed a broad range of responsibilities such as record management, dispatching and tracking journals, reporting to child welfare services, composing discharge summaries, and handling and rescheduling appointments. This consumed, on average, 22.1% of their total work time across all school levels.

Another major part of this category involved meetings, which accounted for 10.9% of the overall time. These meetings included a variety of planned meetings, such as interdisciplinary meetings involving professionals from various fields, and staff meetings attended by those working in school health services and health centers. Meetings held with guardians, known as parents’ meetings in Norway, often took place in the evenings and were recorded under the code for groups involving guardians. These were included in the total tally for activities involving interaction with guardians.

Professional development activities, including courses, professional days, studies, and development work, made up the third largest portion of this category, accounting for 7.2% of the school nurses’ time across all schools.

There were two codes, that each accounted for approximately 5% of the time. Breaks made up 5.2% of the time, a figure consistent across all schools. Preparation and planning time, which was spent preparing for meetings, teaching sessions, and consultations, also represented nearly 5% of the total registered time.

Additionally, there were activities that made up smaller percentages. Aspects such as travel and parking are inherent to school health services, school nurses frequently need to commute between schools and health centers. The code for individual planning referred to the time spent coordinating health services for children or adolescents with complex health challenges, who received services from various health and school sectors.

Waiting and delays refer to the time spent waiting for someone, such as participants at a meeting, while technical problems denote the time spent dealing with technical difficulties, such as issues with computers or other devices. Together, these two categories accounted for 1.1% of the total time across all schools in this study.

In summary, school nurses spent 64% of their total available time across the three school levels on activities that did not involve direct interaction with the users of the school health services (Table 3).

**Table 3:** School nurses’ time related to interaction and noninteraction with children, adolescents and/or guardians

	Activity	10 min Elementary school	%	10 min Middle school	%	10 min High school	%	10 min All schools	%
Activities directly related to interaction with children and/or guardians	Consultation Collaboration guardians Vaccination 1st grade consultation School environment 8th grade consultation Universal teaching Group with guardians Group with pupils 3rd grade consultation	7834	35	4639	35	2271	41	14,744	36
Activities not directly related to interaction with children and/or guardians	Administrative tasks Meetings Courses and studies Breaks Collaboration school Collaboration health Preparation and planning Travel and parking Individual planning Waiting and delays Technical problems	14,791	65	8486	65	3301	59	26,578	64

### Individual variations

Among the 104 school nurses, there was a large variation in the use of the codes. All of them carried out administrative tasks and consultations, whereas for other activities, there was a greater disparity in whether the code was registered as used during these 10 workdays (Table 4). The activities least reported by school nurses were the codes for groups for guardians, followed by the groups for pupils.

There was also a notable variation in the percentage of time spent on the different activities, as shown in Table 4. The basis for the percentage is calculated from each school nurse’s total time used since they worked varying amounts of time over these 10 days. Some dedicated more than 40% of their total time to administrative tasks, consultations, meetings, or courses, whereas others spent minimal or no time at all on these activities during this period. The largest standard deviation was found in consultations, where the spread of time spent by the different school nurses was the greatest. Additionally, a considerable spread was also observed in participation in courses and studies.

**Table 4:** Individual usage of activity codes and variation in percentage

Activity *	Number of nurses using the activity code**	Number of nurses not using the activity code**	Min % ***	Max%***	SD%
Administrative tasks	104	0	6. 7	43. 5	7. 6
Consultation	104	0	1. 6	42. 3	8. 4
Collaboration school	103	1	0. 0	19. 4	3. 0
Breaks	102	2	0. 0	13. 2	2. 5
Meetings	101	3	0. 0	43. 3	6. 9
Collaboration guardians	100	4	0. 0	19. 0	3. 9
Collaboration health	99	5	0. 0	13. 6	3. 2
Travel and parking	94	10	0. 0	11. 3	2. 2
Preparation/planning	92	12	0. 0	21. 6	4. 0
Courses and studies	76	28	0. 0	42. 8	8. 1
Vaccination	76	28	0. 0	23. 5	4. 9
School environment	70	34	0. 0	12. 5	2. 6
Waiting and delays	56	48	0. 0	5. 7	1. 0
Technical problems	47	57	0. 0	3. 8	0. 7
Universal teaching	45	59	0. 0	15. 5	2. 8
Individual planning	30	74	0. 0	12. 9	1. 9
Group with pupils	22	82	0. 0	17. 8	2. 1
Group with guardians	13	91	0. 0	15. 3	2. 2

*Age-determined consultations are not included as they occur only at some stages

**Number of school nurses using the specific activity code or not using the code at all during the period of registration

***Min % represents the school nurses who registered the lowest percentage of usage for this specific activity code. The maximum percentage represents the school nurse that used this code the most. The individual percentages of total school nurse time used during the 10-day period were calculated

## Discussion

The time devoted to directly interacting with pupils and/or their guardians accounted for 36% of the school nurses’ time over a period of ten days, as reported by the school nurses. The remaining time was spent on activities where the school nurses were not physically with children, adolescents, or their guardians. All the activities performed can be found within the Guidelines. However, there is notable variation in the time spent on different tasks and the individual prioritization made by each school nurse.

Time spent directly interacting with pupils, consultations emerged as the most time-consuming activity and were performed by all the school nurses. These consultations represented the time dedicated to dialogue with children and adolescents, both planned and spontaneous ‘drop-in’ sessions, initiated by the pupils themselves. The Guidelines emphasize the importance of school health services being accessible to pupils in their learning environment within the school, functioning as a drop-in service [7]. An essential responsibility of school nurses is to identify children and adolescents experiencing difficulties or showing signs of abnormal development, initiating early interventions when needed [7]. The Guidelines emphasize that young people act spontaneously and may find waiting for an appointment challenging [7]. School nurses hold a unique position in meeting these young people in consultations to promote health and give individual follow- up based on the pupils need. Therefore, maintaining availability for drop-in sessions is essential, as it allows pupils to seek help when needed without lengthy and demanding referral procedures [7, 24]. However, this study does not reveal whether a small number of pupils receive repeated and prescheduled follow-ups, or a high number of different children and adolescents seek support through drop-in consultations.

Collaboration with guardians, another activity classified as direct interaction in this study, is an essential aspect of school health services, although many of these interactions often take place over the phone. Almost all the school nurses collaborated with guardians during this observed period. The percentage of collaboration with guardians decreased for older grades, a trend that can be naturally explained by the age of the children or adolescents and the regulations regarding age and consent. According to the Patient and User Rights Act in Norway, guardians have consent competence on behalf of children aged under 12 years [41]. This implies that school nurses always cooperate with and contact the guardians of children under 12 years of age. The opinions for children and adolescents over 12 years of age should be considered. In general, those aged 16 to 18 have the right to give consent themselves [41].

Group activities, including health education for a class or a group of pupils, constituted a small portion of the total time spent on direct interaction with children and adolescents. A total of 59 school nurses did not use the code for teaching classes, and 82 did not conduct group activities, suggesting that these tasks were not performed by all during this period. The Guidelines emphasize that educating pupils in groups is intended to better prepare children and adolescents to maintain good health and make informed choices for themselves [7]. This is a central part of the school nurses’ responsibilities within the school health services [7]. A central question arises as to why school nurses spend minimal time teaching and performing group activities for pupils. One possible explanation is that school nurses perceive a strong commitment to their work [18] and are highly dedicated to supporting vulnerable children and adolescents [27]. Research suggests that resources are limited, and school nurses often prioritize individual cases and acute follow-ups over health-promoting and universal activities [14, 15, 19, 20, 27]. Deprioritizing children and adolescents who are seeking help might not seem like an option to consider. Furthermore, a Norwegian study highlights that school nurses recognize the need for population based work, however there is a lack of clarity in defining this role within this area [19]. Both the Municipal Health and Care Services Act and Guidelines specify that school nurses should perform health-promoting and preventive teaching in groups or whole classes [7, 10]. However, this is stipulated to be only to the extent that the school requests it [7, 10]. This potentially contradictory and unclear mandate could limit the extent to which school nurses perform these activities. However, conducting group activities could be a more efficient approach than individual consultations, as it allows school nurses to engage with many individuals at once [24, 42]. Moreover, it is sustainable to use resources to strengthen adolescents through preventive and health-promoting group measures, particularly by reducing the need for individual follow-ups [43]. This approach can also contribute to freeing up time for those children and adolescents who require individual consultations [43]. Further research should explore this issue more deeply.

Another code indicating direct interactions with pupils was time spent within the school environment. However, this code was infrequently used. The Guidelines emphasize the importance of school nurses maintaining a presence in the school environment, thereby enabling pupils to independently seek them [7]. In this study, this activity accounted for a small percentage of the total time spent across all schools. The youngest pupils experienced the highest level of school nurse presence in their school environment, a percentage that decreased in middle school, and more in high school. This can be explained by older pupils being more adept at independently seeking out the school nurse and scheduling appointments, whereas younger pupils may need to physically see the school nurse in the school environment to initiate contact. A question to consider is whether an average of nine minutes during a workday constitutes an adequate amount of time for the school nurse to be visible in the environment. This code was used by 70 school nurses, and 34 school nurses were not present in the environment for at least 10 min during the registered period.

The time spent not directly interacting with pupils or guardians accounted for 64% of the total time. Some of the activities were minimal, whereas others occupied a considerable amount of time. Administrative tasks, meetings, and planning took up a large part of the time. Meetings were categorized under a single code and included various types of meetings, with the criterion being prearranged. The Guidelines emphasize that school health services should collaborate with different services both at the municipal level and in primary healthcare [7]. Additionally, there is an emphasis on meetings with schools, where the school nurse should initiate cooperation if it is not already established [7]. This study does not provide information on whom the school nurses met with, the content of the meetings, or the aim of these meetings. However, meetings were the third largest activity performed by school nurses, indicating that it is an activity they spend a large part of their workday on. Future research should examine the content and aim of these meetings in more detail.

Administrative work also occupied a large part of the workdays for the school nurses. In the study from 1957 conducted in New York, it was concluded that school health services could be improved by relieving school nurses of administrative tasks through the use of assistants [31]. This would allow them to spend more time on direct nursing and health promotion activities [31]. This conclusion remains relevant today, suggesting that further studies and development of the service should explore opportunities to delegate some of the paperwork or simplify administrative systems. This could potentially free up time for other activities.

Breaks constituted the fourth largest code in terms of time, not in interaction with the pupils. According to the Norwegian Working Environment Act, an employee with a workday of at least 8 h is entitled to a paid or unpaid half-hour break [44]. If all 104 school nurses took a half-hour break each day during the observed period, this constituted 6.3% of the work time. However, in this study the total time spent on breaks was lower. This finding indicates that school nurses take less break time than recommended. Of the instances where two codes were used simultaneously, five out of the eight multi-codes were used when time was registered as a break alongside another code. This, combined with the fact that school nurses take fewer or shorter breaks than the recommended half hour and often multitask during their breaks, suggests that they may not have adequate time for the breaks they are entitled to. Even though almost every school nurse had at least one break during these two weeks, a proper break is important. The Working Environment Act mandates provisions for facilitating communication and interaction among employees while at work [44]. A break not only gives time for rest and nourishment; it also facilitates socializing among colleagues. This social aspect contributes to a positive work environment, which is linked to avoiding burnout [45]. However, school nurses, who often report high work pressure and understaffing [14–16], may experience shorter or fewer breaks because of these conditions. Consequently, they may choose to multitask, by combining breaks with other work duties.

Overall, our findings reveal that all the activities that school nurses reported performing in the school health service can be found within the Guidelines [7]. However, there are tasks that school nurses spend minimal time on and for some reason, are not prioritized. The Guidelines serve as a job description for school nurses. However, since the Guidelines are normative, describing what is considered professionally acceptable practice [7], school nurses need to make their own choices, prioritize and structure their workday. At the same time, the municipality has significant autonomy to decide on staffing and to organize the service [6, 11]. School nurses report that the current caseload pupil-ratio model is insufficient, as the resources are not enough to fulfill all tasks [14]. Furthermore, reports and studies have highlighted that using a caseload to calculate staffing might not be the most effective approach, as there are many areas that is not counted for [14, 46, 47]. A better workload model should ideally incorporate multiple dimensions, such as the pupil’s health conditions, determinants of health, and individual characteristics of the school nurses [47].

The recent development of the middle school staffing calculator by the Norwegian Directorate of Health represents an advancement toward a more accurate estimation of staffing needs. The calculator outlines the time allocation for activities within the school health service, thereby offering a more comprehensive understanding of staffing [30]. However, comparing this study’s time usage with the calculator is not entirely comparable in all aspects, as some of the activities have different contents. For instance, the time allocated for administrative work is incorporated into various activities in the calculator, whereas in this study, it is distinguished as a separate code. The calculator estimates that administrative work to comprise 10% of a full-time position and includes internal meetings, communication with other services, record keeping (including tracking students’ vaccination status), quality work and deviations, and student supervision, all of which are categorized under different codes in this study [30].

This study indicates a large variation among school nurses in time spent on different activities. The reasons behind each individual’s prioritization are unknown; however, studies suggest that school nurses often have to prioritize among their tasks [14, 15, 17, 18, 27, 48]. A primary aim of the school health services is to provide equal support for all children and adolescents and to operate on evidence based knowledge [7, 12]. However, when activities vary greatly and several tasks are not prioritized, it is important to investigate whether all children and adolescents receive the same level of service. Further research should also focus on the effectiveness of interventions carried out and the capacity school nurses perceive in implementing them. Additionally, there is a need to investigate whether a more equitable regulation between schools and school health services could improve the service. By standardizing the service, the activities performed would become measurable, allowing for adjustment of resources based on needs and evidence-based knowledge.

### Strengths and limitations

This represents the first study where school nurses in the Norwegian school health services registered time spent on their activities. One strength of this study lies in its internal validity [49]. The involvement of four expert groups and three test individuals ensured that the implementation of the time registration proceeded without requiring any adjustments. Furthermore, the high attendance at information meetings, coupled with a low threshold for inquiries, facilitated participants’ understanding of how to register the time log. An additional strength is the real time registration of all activities with pens on paper, thereby eliminating the need for participants to log in and out of digital tools. This method ensured that every 10- minute interval was accounted for and registered.

This study also has some limitations. The external validity of the study presents some weakness [49]. For instance, the school nurses were recruited from three counties in Norway. Therefore, this study may not be representative for all the school nurses within school health services in Norway. In addition, a significant proportion of participants withdrew, totaling 56 school nurses who had initially registered. Many cited illness or capacity issues as their reason for nonparticipation. Consequently, those who completed the study represent a group of school nurses who had the capacity to participate, potentially introducing bias into the results. Another limitation lies in the cross-sectional method, where a two-week period was selected for registration, which might not reflect variations in workload or responsibilities throughout the year. Additionally, the study relied on self-reported data, which can be subject to inaccuracies. Future research should consider using a digital mapping tool and/or objective measures to observe school nurses and document the activities they perform.

## Conclusion

Given that nearly 1,000 days of school health services are registered across various municipalities, this study provides new insights into how school nurses spend their time within Norwegian school health services. Although the study is conducted in a Norwegian context, the methods of describing time usage are transferable to other countries and health sectors.

A notable finding of the study is that 36% of the school nurses’ work time is dedicated to direct interactions with children, adolescents, and their guardians. The remaining 64% are spent on tasks including administration, meetings, planning, and courses. Activities like group guidance and whole class sessions are seldom carried out. Based on the results of this study, there appears to be a potential benefit in simplifying or delegating administrative tasks, potentially freeing up more time for school nurses to focus on their primary responsibilities of health promotion and preventive work.

Further investigations are needed to understand the reasons behind school nurses’ priorities and to assess the potential consequences of the current regulatory framework and the existing resources in school health services. For future development, the introduction of a digital tool is suggested to simplify time studies, contributing to the ongoing improvement of school health services. As this study provides a basis for further exploration, the transition to digital time registration could provide a more detailed and comprehensive monitoring of school health services.

## Electronic supplementary material

Below is the link to the electronic supplementary material.

Supplementary Material 1

Supplementary Material 2

## Data Availability

The datasets used and/or analysed during the current study are available from the corresponding author on reasonable request.

## Abbreviations

Guidelines: National Professional Guidelines for Health Promotion and Prevention Work in Health Centers, School Health Services and Youth Health Clinics
PHN: Public health nurse
