# Supplementary material for: How do school nurses spend their time? A quantitative time study within Norwegian school health services
Source: BMC Nurs. 2025 May 14;24:531. doi: 10.1186/s12912-025-03206-6 (PMC12080160; doi:10.1186/s12912-025-03206-6)
Supplement: Supplementary file 1 — Supplementary Material 1 [file 12912_2025_3206_MOESM1_ESM.pdf]

School nurses' activity list

B: elementary school, U: middle school, VG: high school

Main theme	Task	Code	Example and information
Vaccination	Vaccination	1	All immunization in the child vaccination programme. The time it takes to vaccinate and information about the vaccine. Charting and administrative work around the vaccination are recorded under code 2.
Administrative tasks	Writing journals, letters, referrals, summons, and appointments	2	All administrative tasks such as charting, sending and tracking journals, reporting to the child welfare services, writing discharge summaries, managing and rescheduling appointments.
Routine consultations	1st grade consultation	3.1	The time pupil and guardians are present during the consultation.
	Height, weight measurement and consultation 3rd grade	3.2	The time pupil is present during the consultation.
	8th grade consultation	3.3	The time pupil is present during the consultation.
Consultation	Consultations with pupil/s	4	Consultations with pupil/s.
Guardians	Conversation/phone/meeting with guardians	5.1	Conversation, meeting, and guidance with guardians, with or without the pupil.
	Group guidance	5.2	Parental guidance in a group, universal and on indication. Examples COS, ICDP, DUÅ,
Collaboration with school	Informal conversations with school staff	6	Guidance, conversation, and coordination you have with staff within upbringing/school.
Collaboration with health	Informal conversations with health staff	7	Guidance, conversation, and coordination you have with staff within health sector.
Meetings	Prearranged meetings	8	Various meetings such as interdisciplinary meetings, staff meetings, parent meetings, and planning meetings.
Universal Teaching	Educational School Teaching	9	Universal education and/or preventive measures provided in whole classes or groups.
Groups on Indication	Groups with pupils	10	Groups that are initiated on indication such as living in two homes groups, girl groups, or groups for minorities.
Individual Plan and Coordination Work	Coordinator	11	Time you spend as a coordinator for a pupil who has an individual plan.
Being in the school environment		12	Time you spend in the schoolyard, trips that the school organizes, being in the school corridors.
Preparation and planning		13	Time you use to prepare yourself, such as meetings, teaching, and consultations.
Courses and studies		14	All professional development such as courses and studies.
Waiting and delays		15	Time when you are waiting for something or someone for example, waiting for participants in a meeting.
Technical problems		16	Time you spend on technical problems, such as issues with computer and mobile devices.
Travel and parking		17	Time you spend traveling and parking, for example between schools, courses, or meetings.
Break		18	Lunch or other breaks
Time off/vacation		19	Time off or vacation
Sick		20	Sick leave
Other work than school health services		21	During this period, if you are engaged in activities that are not in the school health services.
Other		22	For any activities that do not fit into the described categories, briefly describe with keyword in the log. Examples: fire drill, relocating an office.
