# Supplementary material for: How do school nurses spend their time? A quantitative time study within Norwegian school health services
Source: BMC Nurs. 2025 May 14;24:531. doi: 10.1186/s12912-025-03206-6 (PMC12080160; doi:10.1186/s12912-025-03206-6)
Supplement: Supplementary file 2 — Supplementary Material 2 [file 12912_2025_3206_MOESM2_ESM.pdf]

Example LOGG

- You need 10 log sheets.
- Blank fields can be used if you have work during the evening.

Time	Code
07:30	
07:40	
07:50	B/U 7
08:00	
08:10	U 2
08:20	
08:30	
08:40	B 3.2
08:50	
09:00	
09:10	
09:20	B 2
09:30	
09:40	
09:50	B 10
10:00	
10:10	
10:20	
10:30	U 4
10:40	
10:50	
11:00	
11:10	
11:20	U 16
11:30	
11:40	
11:50	
12:00	
12:10	U 2
12:20	
12:30	
12:40	
12:50	U 11
13:00	
13:10	
13:20	
13:30	U 5.1
13:40	
13:50	
14:00	
14.10	
14:20	
14:30	
14:40	
14:50	21
15:00	
15:10	
15:20	
15:30	
15:40	
15:50	
16:00	
17:00	B 8
18:00	
